# Supplementary material for: Prevalence and factors associated with comorbid depression and anxiety in patients with diabetes mellitus attending a national referral hospital in Uganda
Source: PLOS Ment Health. 2026 Apr 20;3(4):e0000595. doi: 10.1371/journal.pmen.0000595 (PMC13094950; doi:10.1371/journal.pmen.0000595)
Supplement: Attachment — Submitted filename: Response to Reviewers.docx [file pmen.0000595.s003.docx]

24^th^ March, 2026

Elias Ghossoub,

Academic Editor,

PLOS Mental Health

Dear Sir,

**Re: Response to Reviewers’ comments on the manuscript titled:** “*Prevalence and factors associated with comorbid depression and anxiety in patients with diabetes mellitus attending a national referral hospital in Uganda*”

I extend my gratitude to the team for the initial review and detailed guidance given to improve the manuscript. Herein are the issues raised and the corrections made as discussed with co-authors.

| **Comment** | **Response** | **Page number of the manuscript with track changes** |
| --- | --- | --- |
| 1. The manuscript clearly states that depression and anxiety were correctly diagnosed using the MINI (v7.0.2) however, it states that “the M.I.N.I diagnoses depression and anxiety using the Patient Health Questionnaire-9 (PHQ-9)   and the Generalized Anxiety Disorder-7 (GAD-7)” (line 96), which is inaccurate. Cut off scores are provided for these screening tools however, the MINI does not depend on cut off scores to diagnose the disorders. The results go ahead to dichotomise the anxiety disorders while the GAD-7 does not categorise these but rather only screens for generalised anxiety disorder.  Action required: Clarify the use of the PHQ-9 and the GAD-7 in this study. If they were embedded in the screening, these should be reported independently. Also clarify on the use of cut-off scores. This is essential for validity and reproducibility of the study. | - This statement has been deleted. | Page 4  *Lines 96-102* |
| 2. The abstract (line 34) and the narrative of the results (line 164) report that type I DM is associated with higher odds of comorbidity (aOR 2.48) but tables 2 and 3 report different figures. The reported odds ratios and reference figures in the tables do not match the narrative. There is also inconsistency in the reported odds ratios (crude vs adjusted) in the abstract and the results. | - The discrepancy has been revised as recommended in the abstract. | Page 2  *Lines 32-36*  Page 8  *Line 168* |
| 3. Extremely wide confidence intervals appear in table 3 which raises concerns on the model stability. | - With only 223 participants, the sample size was small relative to model complexity since the outcome has three categories (*neither condition, depression or anxiety, comorbidity*) and several predictors. Multinomial logistic regression requires enough cases in each outcome category. Since some variables have very few observations in the outcome categories the confidence intervals widen. |  |
| 4. Ensure all reference categories are clearly stated. | - This has been revised as recommended | Pages 8-10  *Lines 166-190* |
| 5. Clarify the reported estimated and harmonise these throughout the text (abstract and results). | - This has been revised as recommended | Page 2  *Lines 32-36*  Page 8  *Line 168* |
| 6. Recheck the regression models. | - This has been rechecked as recommended |  |
| 7. When considering blood pressure, this was categorised as “high and normal” in tables 2 and 3, which was measured during the visit (line 90). In the discussion section (line 232), this is discussed as “Patients with hypertension” which shows an inconsistency in the reporting. | - This has been revised as recommended | Page 12  *Lines 238-247* |
| 8. There is repeated interpretation of odds ratios as “increased likelihood” (line 208), “less likely” (line 241) which imply causal inference yet this cannot be ascertained from a cross-sectional study. Using words like “more associated with” would be more appropriate. | - Odds ratios quantify how strongly patient characteristics are linked to comorbid depression and anxiety, but they do not prove cause-and-effect. This is mentioned in the limitations in lines 279-282 Risk of comorbid depression and anxiety is best measured by risk ratios. The ORs describe the “likelihood” which means probability of comorbid depression and anxiety happening. We use likelihoods to make the results more clinically meaningful. - Revisions have been made in the methods to clarify this. | Page 5  *Lines 129-130, 136-137*  Page 12  *Lines 279-282* |
| 9. Clarify whether individuals with high blood pressure or hypertension were considered given that the two are not synonymous. | - This has been revised as recommended | Page 12  *Lines 238-247* |
| 10. Clarify the association of the factors with the outcome. | - Odds ratios quantify how strongly patient characteristics are linked to comorbid depression and anxiety, but they do not prove cause-and-effect. - Revisions have been made in the methods to clarify this. | Page 5  *Lines 129-130, 136-137*  Page 12  *Lines 277-279* |
| 11. Interviewer training is not mentioned. It would be more informative to include it. | - This has been revised as recommended | Page 4  *Lines 84-85* |
| 12. The tables would benefit from captions. | - This has been revised as recommended | Page 7  *Line 153*  Page 9  *Line 182*  Page 9  *Line 192* |
| 13. Figure 1 would also benefit from a caption explaining the colour codes | - A description of the figure has been included in the results. | Page 7  *Lines 160-162* |
| 14. Quality of Life domains are discussed as predictors however, these can also be a consequence of the comorbidity of depression and anxiety. This would benefit from a clarification on this. | - This has been clarified. | Page 8  *Lines 173-177*  Page 10  *Lines 184-186* |

Yours Sincerely,

**Sophia Balinga**, MBChB (Mak), MMED (Mak)

Department of Psychiatry, College of Health Sciences, Makerere University

[sophiebalinga123@gmail.com](mailto:sophiebalinga123@gmail.com)
